# Protein Databases Related to Liquid–Liquid Phase Separation

**DOI:** 10.3390/ijms21186796

**Published:** 2020-09-16

**Authors:** Qian Li, Xi Wang, Zhihui Dou, Weishan Yang, Beifang Huang, Jizhong Lou, Zhuqing Zhang

**Affiliations:** 1 College of Life Sciences, University of Chinese Academy of Sciences, Beijing 100049, China; liqian175@mails.ucas.edu.cn (Q.L.); wangxi192@mails.ucas.ac.cn (X.W.); douzhihui19@mails.ucas.ac.cn (Z.D.); yangweishan19@mails.ucas.ac.cn (W.Y.); huangbeifang19@mails.ucas.ac.cn (B.H.); jlou@ibp.ac.cn (J.L.); 2 Key Laboratory of RNA Biology, CAS Center for Excellence in Biomacromolecules, Institute of Biophysics, Chinese Academy of Sciences, Beijing 100101, China

**Keywords:** liquid–liquid phase separation, protein, databases, membraneless organelles, condensates

## Abstract

Liquid−liquid phase separation (LLPS) of biomolecules, which underlies the formation of membraneless organelles (MLOs) or biomolecular condensates, has been investigated intensively in recent years. It contributes to the regulation of various physiological processes and related disease development. A rapidly increasing number of studies have recently focused on the biological functions, driving, and regulating mechanisms of LLPS in cells. Based on the mounting data generated in the investigations, six databases (LLPSDB, PhaSePro, PhaSepDB, DrLLPS, RNAgranuleDB, HUMAN CELL MAP) have been developed, which are designed directly based on LLPS studies or the component identification of MLOs. These resources are invaluable for a deeper understanding of the cellular function of biomolecular phase separation, as well as the development of phase-separating protein prediction and design. In this review, we compare the data contents, annotations, and organization of these databases, highlight their unique features, overlaps, and fundamental differences, and discuss their suitable applications.

## 1. Introduction

**Figure 1:**
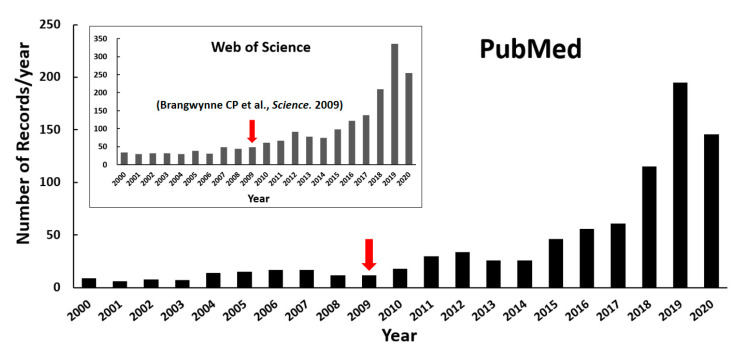
Number of publications on protein LLPS investigation over the past twenty years (until the end of August in 2020). The retrieval was performed with the keyword combinations “((liquid−liquid phase separation) OR (liquid−liquid phase transition)) AND (protein)” from NCBI PubMed as well as Web of Science (inserted figure). The red arrows highlight Brangwynne CP and coworkers’ publication shown in 2009.

Biomolecules within intracellular compartments cooperate spatiotemporally in controlling efficient and precise biochemical reactions in cells. These compartments can be roughly divided into membrane-bounded organelles and membraneless ones, with distinct structural organizations. Unlike the classic organelles bound by bilayer lipid membranes, the membraneless compartments have no membrane and are, therefore, called membraneless organelles (MLOs) or biomolecular condensates, such as the Cajal body in the nucleus, the stress granule (SG) and P-body (PB) in the cytoplasm, the nuage in the germ cell, receptor clusters, and the pyrenoid matrix [1,2]. It is widely appreciated that the formation of MLOs is regulated by liquid–liquid phase separation (LLPS) of biomolecules since Brangwynne CP and coworkers’ first analysis of liquid droplets in Drosophila embryos in 2009 [3]. As a result of growing research interests, the publications on LLPS of biomolecules have increased explosively in recent years, as shown in the statistical plot in Figure 1.

Biomolecular LLPS is a reversible molecular process of certain proteins and/or nucleic acids being condensed into a dense phase coexisting with a dilute phase [4]. The physicochemical properties of liquid condensates suggest LLPS processes perform a variety of biological functions, as reviewed in Alberti and coworkers’ paper [5]. Biomolecular LLPS can be regulated by mutations or post-translational modifications (PTMs) of proteins, which might be implicated in a range of incurable neurodegenerative diseases such as amyotrophic lateral sclerosis (ALS) [6,7], frontotemporal dementia (FTD) [8], and Alzheimer’s disease (AD) [9]. It implied that LLPS provides a new angle for researchers to inspect these diseases and various cellular processes.

Given that many physiological and pathological functions have been discovered to be associated with LLPS processes, there is a pressing need to identify the underlying driving mechanism [10,11,12]. Many proteins and nucleic acids have been found to be able to undergo LLPS both in vivo and in vitro [13,14,15,16,17]. Multivalent weak interactions are fundamentally deemed as the main driving force for LLPS [18,19], which are characterized as multisite dynamic physical crosslinking among biomolecular chains via weak binding forces such as electrostatic, cation–π, π–π, hydrogen bonding, and hydrophobic interactions [20,21,22]. Multivalent weak interactions can generally occur in proteins between multiple folded domains or between multiple interacting motifs in intrinsically disordered regions (IDRs) or between the both of them [23], as well as between proteins and RNAs/DNAs [24,25,26]. No matter how, intrinsically disordered proteins (IDPs) or long IDRs play essential roles in driving the LLPS process [27]. They are highly flexible and lack stable 3D structures and harbor repetitive linear motifs or low-complexity regions (LCRs), thus possess great advantage to form transient multivalent weak interactions or provide the flexibility of systems [28]. The sequence length of IDRs, as well as the sequence pattern, which can be modified by residue mutation, repeating certain motifs or PTMs, could mediate the phase separation propensity of proteins [2,29]. How the various IDPs or IDRs and their modifications regulate the formation of MLOs and perform their biological functions have attracted the attention of researchers recently [30,31].

Protein can phase-separate on its own or with other molecules. Those required for the formation of condensates are referred to as scaffolds, while others that partition into condensates without playing an essential role are called clients [28]. Due to the promiscuous interactions of IDRs, some proteins may contribute to distinct condensates as scaffolds or as clients. The phase separation process may be regulated by other proteins, RNA/DNA, or molecules such as ATP, which are coined as regulators in some publications [32,33,34]. In addition, environmental parameters such as the concentrations of protein, nucleic acid, and salt, as well as the pH, pressure, and temperature of the system have been demonstrated to be able to regulate the LLPS process [28,29]. In some situations, changes in molecular features or cellular environment may further transform liquid-like condensates into gel- or solid-like states [35,36,37]. These various influenced factors suggest that the phase behavior of biomolecules can be regulated through multiple aspects for normal cellular processes, adaptions, and dysfunctions [38].

The intensive investigations in the phase separation of biomolecules provide a data foundation for a more comprehensive and deeper understanding of LLPS in cell biology. Around 40 MLOs have been suggested to be organized via phase separation in eukaryotes, bacteria, and viruses [39], and several studies have reviewed the components and functions of MLOs [40,41,42,43]. Recently, a couple of databases covering the function and formation mechanism of condensates, experimental information, and localization information of LLPS-related proteins such as LLPSDB, PhaSePro, PhaSepDB, DrLLPS, RNAgranuleDB, and HUMAN CELL MAP, have been released. Together, they provide researchers a comprehensive overview and undoubtedly serve as valuable resources. In this review, we briefly describe and compare the content, annotation focus, differences, and overlap of these databases and their applicability to experimental and computational LLPS studies.

## 2. Databases Related to LLPS

Six LLPS-related databases, four containing proteins from direct LLPS studies and two constructed based on proteome identification, are described here. The former includes LLPSDB [44], PhaSePro [45], DrLLPS [39], and PhaSepDB [46]. Within all or part of them, deposited proteins are validated by LLPS experiments. Each database provides the basic information of recorded proteins, as well as their structural and functional annotations. The phase behavior information of proteins is also deposited in each database in more or less detail. The latter includes RNAgranuleDB [47] and HUMAN CELL MAP [48], in which the proteome of organelles (specifically MLOs SG and PB in RNAgranuleDB) is curated. A general summary for each of the six databases is shown in Table 1.

### 2.1. LLPSDB

LLPSDB (http://bio-comp.ucas.ac.cn/llpsdb or http://bio-comp.org.cn/llpsdb) is the first released database designed specifically for proteins undergoing LLPS that have been validated by experiments in vitro [44]. Currently, 273 individual proteins and 1175 entries have been deposited. It is the only database incorporating both natural and designed proteins. An entry in LLPSDB is defined by specific protein sequence and nucleic acid type in the system. Therefore, although both wild-type FUS and its cleaved low complexity region (LCR) can undergo LLPS, they belong to different entries in LLPSDB. Condensates formed by the same protein(s) with 15 nt RNA and 30 nt RNA also belong to different entries. All the deposited data were grouped based on protein type (natural/designed), main component type (only protein(s)/protein(s) + RNA/protein(s) + DNA), or main component number (one/two/more). The detailed functional and structural information of wild-type or designed protein is recorded in the protein details page, which can be accessed through the “protein type” classification on the browsing page or the linkage (“Link to protein” in the “General information” part) on the entry page. The functional description provided in LLPSDB integrates information retrieved from UniProt [49] and the literatures. IDRs and LCRs based on related databases or algorithms are visualized in the protein details page. Crosslinking to other functional related databases—Uniprot [49], MobiDB [50], DisProt [51], OMIM [52], IDEAL [53], FuzzDB [54], and AmyPro [55]—are provided. In addition to being able to access the entries through the “Browse” page, users can search the database by specific keywords or perform a protein sequence blast via the “Search” page. All data in LLPSDB can be downloaded from the “Download” page, according to the three different classifications.

A unique feature of LLPSDB is that it includes the specific experimental conditions adopted in each LLPS system. The protein sequence, modifications (including cleaved, fusion, motif repeats, mutation, and PTM), as well as experimental parameters such as protein and nucleic acid concentrations, salt concentration, crowding agent concentration, pH, temperature, and pressure, are clearly listed in each entry. Furthermore, the database also includes those comparative “negative” situations, where “no” phase separation was detected in the specific experimental condition in the corresponding system. Meanwhile, more than 200 phase diagrams in the corresponding literature, which provide the critical phase separation condition of LLPS systems, are also recorded in LLPSDB. Although it is designed specifically for proteins undergoing LLPS in vitro, LLPSDB additionally records whether there are corresponding in vivo (or in cell) experiments in the corresponding literature for each system. However, it does not include proteins with only in vivo experiments or those only identified in MLOs but without detailed experimental conditions of LLPS.

### 2.2. PhaSePro

PhaSePro (https://phasepro.elte.hu) is a novel database in which proteins verified to drive phase separation in vivo and/or in vitro are manually curated [45]. It contains 121 proteins, with 109 from eukaryotes, 5 from bacteria, and 7 from viruses. In each entry, very detailed LLPS annotations of the corresponding protein are carefully and manually summarized based on all currently available LLPS studies or existing databases. In addition to some general information of the protein being provided, such as localization and species, the molecular features including IDR prediction by IUPred [56], domain predictions by PfamScan [57], PTMs from PhosphoSitePlus [58], as well as the cartoon-style PDB structural view for the corresponding LLPS protein regions, are also incorporated into a graphical representation. In “Extended LLPS information”, the protein regions that have been demonstrated to drive LLPS, the partners, the molecular interaction types, the determinants of phase separation, and droplet property, as well as the annotations on regulation and related disease, are listed in each entry page. The functional description and experimental information of LLPS are also recorded in the form of free-text, together with the supporting literature references. Data in PhaSePro can be accessed via a keyword search on the “Home” page or the “Browse/Search” page or by directly browsing all entries on the “Browse/Search” page. PhaSePro provides several options for users to download the data, including downloading selected entries from the “Browse/Search” page and downloading the full database in JSON, TSV, or XML format from the “Download” page.

In addition to detailed LLPS information such as protein regions driving LLPS and the molecular interaction types mentioned above, another outstanding feature of PhaSePro is that it introduces LLPS-specific controlled vocabularies (CVs) that are custom-built based on the literatures, including the functional, molecular, and experimental information of the protein which drives LLPS. Four distinct CVs have been developed in these aspects: (i) 8 classes of the functional roles of membraneless organelles/granules in the cell, (ii) 19 terms for the different molecular interaction types, (iii) 6 terms to describe the molecular determinants and mechanisms, and (iv) 7 terms of experimental observations supporting the liquid state of condensates. Using CVs to standardize the annotations in this database greatly reduces the redundancy of related information and helps the interpretation of each entry.

### 2.3. PhaSepDB

PhaSepDB (http://db.phasep.pro/) currently contains 2914 non-redundant proteins localized in more than 30 MLOs [46]. It includes the known 352 LLPS-associated proteins extracted from published literature, 378 potential proteins reviewed from UniProt according to their subcellular locations, as well as 2516 proteins with localization identified by high-throughput experiments, including organelle purification, proximity labeling, immunofluorescence image-based screening, and affinity purification. Therefore, in this database, those proteins localized in specific membraneless organelles with no direct LLPS investigations are considered LLPS-related. For each entry, PhaSepDB provides the information of the protein, such as species, localization, IDR content, supporting literature, as well as functional description, cell line, and some experiment details and notes, with original sentences from literatures. The data can be accessed either through different sources, as described above on the “Data Sources” page, or through a keywords search or specific membraneless body location in the form of graphical navigation on the “Home” page. Most of the data can be downloaded from the “Download” page according to three different sources.

It is worth noting that PhaSepDB provides various bioinformatic analyses of sequence properties and displays each of them using an easily interpreted per-residue plot. The analysis integrates the results of IDR prediction by ESpritz [59], prion-like sequence prediction by PLAAC [60], electrostatic interaction prediction by Pi-Pi [61], as well as charged/hydrophobic residue distribution analysis by CIDER [62]. It also contains post-translational modifications (PTMs) [63], secondary structure annotations, and domain and compositional bias annotations. The molecular properties analysis in PhaSepDB is also provided for all human proteins to help the identification of potential LLPS proteins.

### 2.4. DrLLPS

DrLLPS (http://llps.biocuckoo.cn/) is a gene-centered database and currently holds the largest amount of data [39]. In total, it contains 437,887 proteins in 164 eukaryotes, including 150 scaffold proteins, 987 regulators, and 8148 potential client proteins manually curated from published literature, and their orthologs, which are considered potential LLPS-associated proteins identified via a genome-wide detection by protein sequence blast. The scaffolds are defined as the drivers of LLPS; the regulators refer to proteins that have not been identified to undergo LLPS but are known to be involved in regulating the stability and formation of MLOs and/or liquid droplets; the clients here mean those proteins that are co-complexed or co-localized with scaffolds but are not known to be indispensable for the formation of condensates. Data can be accessed through three categories: 40 biomolecular condensates belonged to five superclasses, including in vitro droplet, nucleus, cytoplasm, germ cell, and others; LLPS types—scaffolds, regulators and clients; species which mainly include proteome sets of 68 animals, 50 plants, and 46 fungi. There are also various search options provided on the “Search” page for users to access the datasets, including simple search, batch search, and advance search by inputting keywords. Meanwhile, a blast search for protein sequence is also offered. The data of the known 9285 LLPS−associated proteins detected by experiments can be downloaded from the “Download” page in TXT format. However, for the full datasets of all proteins from both experiments and computations, the protein/DNA sequences and annotations can only be downloaded in groups according to protein species and annotation sources.

The annotations for each protein in DrLLPS include basic information such as Ensembl [64]/UniProt [49]/GeneBank [65]/RefSeq [66] accession numbers, functional descriptions, and protein/nucleotide sequences. DrLLPS also presents brief descriptions of protein roles in LLPS, localizations, effects of partners, experimental analysis descriptions in vitro and/or in cells, as well as primary supporting references. In addition, it provides very comprehensive molecular feature annotations from 110 widely-used public resources for 28,024 known and potential LLPS-associated proteins in eight model species, which cover 16 aspects, including IDR prediction, domain annotations, PTMs, genetic variations, cancer mutations, protein 3D structures, and subcellular localizations. Although most of the information is computationally predicted and has not been detected in LLPS experimental studies, it brings researchers substantial useful information and will assist in further related investigations.

### 2.5. RNAgranuleDB and HUMAN CELL MAP

RNAgranuleDB (http://rnagranuledb.lunenfeld.ca) [47] and HUMAN CELL MAP (https://cell-map.org/ or https://humancellmap.org/) [48] are two databases that are particularly focused on the proteome of organelles, in which those deposited proteins in MLOs are related to LLPS.

RNAgranuleDB provides a comprehensive summary of SG and PB components, and, in total, 4385 mammalian proteins (from human, mouse, and rat) are collected. All these proteins have been manually curated from 122 peer-reviewed publications and identified by either high-throughput experiments or low-throughput approaches. They are categorized into 4 tiers, weighted according to the degree of experimental support for the residence in SGs or PBs. Among them, proteins in the Tier 1 group have the highest confidence to be considered SG or PB proteins, while the Tier 4 group primarily consists of RNA-binding proteins with no specific evidence of association with SGs or PBs. RNAgranuleDB analyzes the potential LLPS capability of these proteins by six first-generation predictors based on sequence features associated with aggregation or phase-separation properties reviewed in ref [67]. It was expectedly found that proteins in the higher tier groups contain a larger fraction of proteins, showing significant LLPS matches for all of the sequences with the predictions. Users can access data via direct browsing or a search on the home page and are allowed to download all tiers or only the Tier 1 dataset through the “Export” window on the RNAgranuleDB webpage.

HUMAN CELL MAP is another database in which proteins that are not only in MLOs but also in membrane-bound organelles identified in HEK293 cells are curated based on the proximity-dependent biotinylation approach BioID. A total of 192 markers (baits) and 4145 high confidence prey proteins are collected, generating an interactive map that elaborates different sets of associated proteins in the cell. In addition to general information, the enrichment for expected domains and motifs, as well as the GO terms for each organelle, is also analyzed. HUMAN CELL MAP is not specifically targeted at biomolecule phase separation, but a number of proteins in the database have been identified to be localized in various MLOs, which means they may drive or be related to phase separation to form these MLOs. According to the interactive map within the database, the associated proteins of searched prey will certainly expand the biofunctional understanding of the corresponding MLOs. For this database, there are three ways provided on the “Explore” page to access the dataset: browsing by a 2D interactive cell map, browsing by specific organelles or its components, and searching by the official gene symbol or synonym. All the baits and preys, as well as the bait–prey pairs, can be downloaded on the “Download” page in TXT format.

## 3. Comparison of the Databases

**Figure 2:**
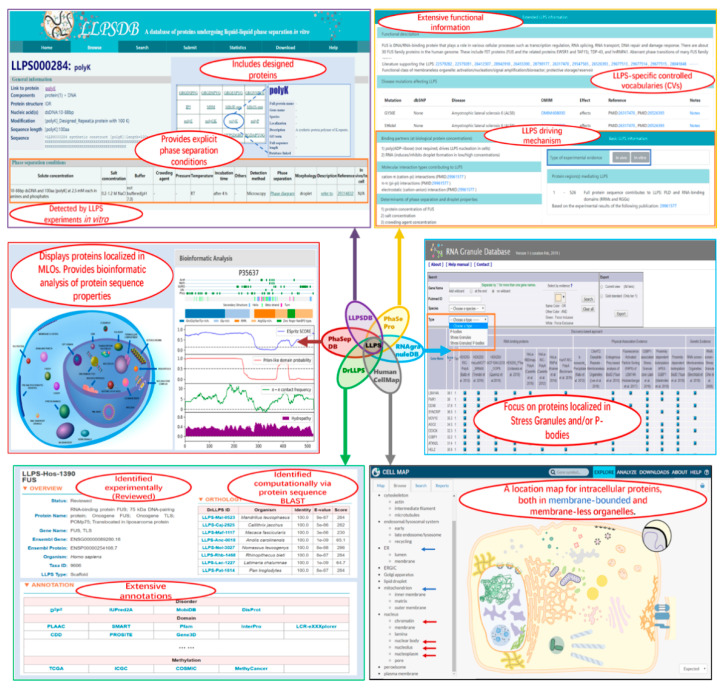
Screenshots of some webpages of the six related databases. In each squared screenshot, the unique features of the corresponding database are shown in red font text within the ellipse region(s).

These six databases provide valuable information on the LLPS system and MLO components. They overlap each other to different extents. Meanwhile, each database is designed for specific aims and has unique features (as shown in Figure 2). We compare the databases on the following aspects: data groups and sources, annotations, and suitable applications.

Data collected in these databases overlap each other to different extents, as shown in Table 2, which can be mainly grouped into two classes: one for proteins undergoing or involving LLPS that have been validated directly by in vivo and/or in vitro experiments, and the other for proteins identified or predicted to be components of known MLOs or biomolecular condensates. Currently, more than one hundred proteins have been verified to undergo or involve LLPS directly. Four resources—LLPSDB, PhaSePro, PhaSepDB, and DrLLPS—collect them. All proteins in LLPSDB have been verified to undergo (or NOT undergo) LLPS in vitro on their own or with other proteins or nucleic acids. PhaSePro focuses on proteins driving LLPS with explicit in vitro and/or in vivo experimental evidence. The difference in proteins within them arises from that LLPSDB contains designed proteins and some deposited proteins, which may not function as drivers but as clients or regulators in those multiple-component systems, while PhaSePro includes those proteins validated to undergo LLPS in vivo but not in vitro. In addition to the first data group, PhaSepDB and DrLLPS also incorporate the second class of data. PhaSepDB includes the proteins localized in membraneless compartments that are recorded in UniProt or identified by high-throughput experiments. In DrLLPS, proteins in various biomolecular condensates with experimental identification, are collected and classified. Moreover, based on genome-wide detection via protein sequence blast, the orthologs of both data groups in 164 eukaryotes are also deposited. The numbers of proteins predicted or identified based on UniProt are listed in Table 1. RNAgranuleDB provides the currently available compositions of SG and PB proteomes, and HUMAN CELL MAP is curated for protein components in both membrane-bound and membraneless compartments from the HEK293 cell. The data in the latter two databases are experimentally validated, although the proteins within them have different confidence levels. The overlapped number between the last four databases in Table 2 means that the data may come from the same literature (except HUMAN CELL MAP) or the MLO localization of proteins identified by different approaches.

Although all the databases provide general information of deposited proteins, such as protein name, species, localization, function, PMID, and short description from the literature, the annotations of experimental details, as well as molecular properties analysis in each of them, are various and have their own emphases. LLPSDB provides in-depth annotations describing the verified phase behavior of the system in each entry, exhaustive molecular modifications such as cleaving, mutation, and PTMs for specific protein constructs, and corresponding explicit phase separation conditions, as well as phase diagrams. For sequence properties, it includes IDR and LCR predictions for each wild-type protein. PhaSePro contains a broader array of functional and disease information of LLPS. It also provides the LLPS driving regions, molecular interaction types, as well as detailed LLPS experimental information in free-text form. The proposed LLPS-specific CVs are applied to standardize the descriptions of functional roles, LLPS experimental information, as well as molecular interaction type or determinants for the protein in each entry. These CVs reduce the redundancy of information in the database and aid the interoperability and computational analyses of the database, which may provide the foundation of data standards in the rapidly expanding field of biomolecular LLPS. Structure-related annotations in PhaSePro are more abundant, including not only the predicted IDRs but also the PTMs, sequence variants, and 3D structures in visualization. PhaSepDB specifically provides useful sequence analysis such as PTMs, secondary structure distribution, electrostatic interaction, and hydrophobic residue distribution, displaying each by an easily interpreted per-residue plot. The graphical navigation on the home webpage makes it very convenient for users to find the MLO information they are interested in. DrLLPS includes the most comprehensive structure-related annotations. It integrates 110 widely-used public resources to describe the protein structural and functional features from 16 aspects, with each aspect summarized by no less than two kinds of resources. For RNAgranuleDB and HUMAN CELL MAP, they both focus on the proteome of organelles; therefore, their annotations lack detailed information of LLPS but include more evidence of localization in MLOs with experimental identification, which will extend the understanding of MLOs and LLPS function.

These databases are complimentary, and, together, they provide valuable and comprehensive resources to facilitate the research of biomolecular phase separation and cellular organization, not only in the experimental aspect but also in the development of theory and prediction algorithms. The proteins deposited in LLPSDB and PhaSePro are all verified by LLPS experiments, which constitute a high-quality training set for the development of new methods to identify novel LLPS proteins. In LLPSDB, specific protein constructs with corresponding specific experimental conditions for LLPS will further help researchers to understand how the phase behavior of protein is sensitive to the environment in order to design algorithms for predicting the phase separation propensity of new proteins. Recently, a predictor of LLPS protein (PSPredictor, http://www.pkumdl.cn/PSPredictor) based on machine learning was developed [68], using the datasets in LLPSDB as a training set. It achieved a fairly high prediction accuracy and outperformed other reported prediction tools so far, which are all based on specific protein sequence features [61,67,69]. The well-summarized structural, functional, and detailed experimental information provided in PhaSePro makes it very useful for researchers to find complete and systematic knowledge of LLPS proteins. PhaSepDB and DrLLPS include more proteins related to LLPS that have been verified by experiments or likely localized in MOLs or biomolecular condensates. The extensive molecular property analysis within them could provide helpful information to understand if they might be potential proteins to undergo or regulate LLPS in future investigations. The large number of orthologs and their annotations recorded in DrLLPS make it specifically useful for analyzing LLPS from an evolutionary perspective. Taken together, a suitably combined application of these databases would definitely advance a deeper understanding of LLPS in cells.

## 4. Summary

Investigations on biomolecular LLPS or the formation of biomolecular condensates have grown fast in recent years. A number of databases have been timely constructed to curate the mounting generated data, which will undoubtedly make advances in the research of biomolecule phase separation. Here, six recently released protein databases related to LLPS—LLPSDB, PhaSePro, PhaSepDB, DrLLPS, RNAgranuleDB, and HUMAN CELL MAP—are discussed and compared. Although the data within them are overlapped to a certain extent, the organization and annotations in each of them have their own focuses and unique features. We believe this thorough review of these databases will provide researchers a general perception and help users to utilize these resources efficiently.

## Figures and Tables

**Table 1:** Overview of six databases related to liquid–liquid phase separation (LLPS).

Databases	Organization	Data Contents	Data Sources	Outstanding Features	Availability	Ref.
LLPSDB	Entries are defined by specific protein and/or nucleic acid constructs. Classified by (i) Protein type (natural, designed) (ii) Components type (protein(s), proteins(s) + RNA, protein(s) + DNA) (iii) Components number (one, two, more)	273 proteins 1175 entries	Validated by LLPS experiments in vitro	Including natural and designed proteins Provides exhaustive molecular modifications, including fusion, cleaved, mutation, repeat, and PTMs, that are detected experimentally for specific protein constructs Provides explicit phase separation conditions (environmental parameters) and more than 200 phase diagrams	http://bio-comp.ucas.ac.cn/llpsdb or http://bio-comp.org.cn/llpsdb	[44]
PhaSePro	Entries are defined by specific proteins.	121 proteins (109 from eukaryotes, 5 from bacteria, and 7 from viruses)	Validated by LLPS experiments in vitro and/or in vivo	Provides LPS driver region(s) and molecular interaction types contributing to LLPS, validated by experiments for each protein Introduces LLPS-specific controlled vocabularies (CVs) to annotate the functional, molecular, and experimental information of each protein Provides a broader array of structural, functional, and disease information	https://phasepro.elte.hu	[45]
PhaSepDB	Entries are defined by specific proteins. Classified by (i) Data sources (reviewed, UniProt reviewed, high throughput) (ii) Location and organelle (more than 30 MLOs)	2914 proteins (352 are detected by LLPS experiments; 378 are reviewed from UniProt according to protein localization in MLOs; 2572 are identified to be localized in MLOs based on high throughput experiments)	Validated by LLPS experiments Localized in membraneless compartments through UniPort review and high throughput experimental validation	Entries can be browsed through specific MLO locations in the form of graphical navigation on its home page Provides various bioinformatic analysis of the sequence properties such as PTMs, secondary structure distribution, the electrostatic interaction, and hydrophobic residue distribution and displays the results by an easily interpreted per-residue plot Provides sequence analysis of other human proteins	http://db.phasep.pro/	[46]
DrLLPS	Entries are defined by specific genes. Classified by (i) Condensates (in vitro droplet, nucleus, cytoplasm, germ cell, Others) (ii) LLPS types (scaffold, regulator, client) (iii) Species (animals, plants, fungi)	437,887 proteins in 164 eukaryotes (9285 are identified experimentally, 428,602 are identified computationally via protein sequence blast)	Validated by experiments of LLPS or membraneless compartments identification Identified computationally via the protein sequence blast	Holds the largest amount of data Includes the most comprehensive structure-related annotations from 110 public resources covering 16 aspects	http://llps.biocuckoo.cn/	[39]
RNAgranuleDB	Entries are defined by specific proteins. Three hierarchical levels: (i) Experiment design (discovery-based approach, candidate-based approach) (ii) Evidence type (cell biological, physical, genetic) (iii) Specific assay or dataset	4385 proteins (368 proteins were assigned to Tier 1 with highest confidence SG-PB proteins, 475 to Tier 2, 428 to Tier 3, and 3114 to Tier 4)	Localized in stress granule and P body, validated by experiments	All proteins are categorized into 4 tiers weighted according to the degree of support it provides for protein residence in SGs or PBs. Proteins are analyzed by the prediction of six first-generation LLPS predictors Lacks detailed information on LLPS	http://rnagranuledb.lunenfeld.ca	[47]
HUMAN CELL MAP	Entries are defined by specific genes. Classified by organelle type (membrane-bound or membraneless)	4145 proteins	Localized in membrane-bound or membraneless organelles through identification based on experiments combined with analysis.	Summarizes for each compartment the enrichment of expected domains and motifs as well as GO-terms Provides channels to analyze spatiotemporal correlations between proteins in different organelles Lacks detailed information on LLPS	https://cell-map.org/ or https://humancellmap.org/	[48]

**Table 2:** Overlapped protein numbers between the six databases related to LLPS. (The numbers of overlapped proteins between any two databases were obtained though “UniProt ID” except for RNAgranuleDB. For the overlapped proteins between RNAgranuleDB and other databases, “gene name” was used for comparison. The diagonal blue number shows the number of proteins deposited in each database (for DrLLPS, the potential orthologs were not included), which is somehow slightly different from that reported in the corresponding paper for PhaSepDB, DrLLPS, and RNAgranuleDB, probably due to correction after the databases’ release.))

	LLPSDB	PhaSePro	PhaSepDB	DrLLPS	RNAgranuleDB	HUMAN CELL MAP
**LLPSDB**	***273***					
**PhaSePro**	**65**	***121***				
**PhaSepDB**	**94**	**82**	***2957***			
**DrLLPS**	**115**	**83**	**1520**	***9281***		
**RNAgranuleDB**	**75**	**56**	**491**	**2440**	***4386***	
**HUMAN CELL MAP**	**45**	**35**	**1056**	**1825**	**2519**	***4424***

## Abbreviations

LLPS	Liquid−liquid phase separation
MLOs	membraneless organelles
SG	stress granule
PB	P-body
PTMs	post-translational modifications
ALS	amyotrophic lateral sclerosis
FTD	frontotemporal dementia
AD	Alzheimer’s disease
IDRs	intrinsically disordered regions
IDPs	intrinsically disordered proteins
LCRs	low-complexity regions
CVs	controlled vocabularies
